# Extra-Nodal Lymphomas of the Head and Neck and Oral Cavity: A Retrospective Study

**DOI:** 10.3390/curroncol29100566

**Published:** 2022-09-29

**Authors:** Alfonso Sorrentino, Francesco Ferragina, Ida Barca, Antonella Arrotta, Maria Giulia Cristofaro

**Affiliations:** 1 Department of Neurosciences, Reproductive and Odontostomatological Sciences, Unit of Maxillofacial Surgery, University Hospital of Naples “Federico II”, 80131 Napoli, Italy; 2 Department of Experimental and Clinical Medicine, Unit of Maxillofacial Surgery, “Magna Graecia” University, Viale Europa, 88100 Catanzaro, Italy; 3 Department of Medicine and Surgery Sciences, Magna Graecia University, Viale Europa, 88100 Catanzaro, Italy

**Keywords:** lymphoma, head and neck neoplasm, non-Hodgkin’s lymphoma, extra-nodal non-Hodgkin’s lymphoma, oral non-Hodgkin’s lymphoma

## Abstract

*Disease Overview*: Lymphomas, both Hodgkin’s and non-Hodgkin’s lymphomas, are one of the most common cancers in the head and neck area. The extra-nodal variant of lymphoma is rare, but it is the most common non-Hodgkin’s lymphoma (ENHL). Furthermore, it is difficult to diagnose due to its non-specific clinical and radiological features, which can mimic other benign or malignant clinical manifestations. *The study*: This retrospective study involved 72 patients affected by head and neck ENHL in the period between 2003 and 2017. All patients underwent a diagnostic-therapeutic procedure according to the guidelines, and a 5-year follow-up. Based on the location of the swelling at the time of diagnosis, patients were divided into two groups: oral and non-oral ENHLs. Statistical analysis was performed using Kaplan–Meier analysis with the log-rank test. In addition, Fisher’s exact test was applied to the two groups to evaluate and compare variances (the acceptable significance level was set at *p* < 0.05). *Conclusion*: ENHL with oral localization is much more aggressive than ENHL with non-oral localization, with a death rate of 40% (versus 4.76 for the non-oral one). In fact, between the two groups, there is a statistically significant difference in mortality, with a *p*-value of 0.0001 and 0.0002, respectively.

## 1. Introduction

Lymphomas are malignant neoplasms of lymphocyte cell lines, accounting for approximately 14% of all malignant neoplasms of the head and neck. They are divided into two subtypes, Hodgkin’s lymphomas (HLs) and non-Hodgkin’s lymphomas (NHLs), with differences in histology, clinical features, and prognosis. HL often appears as a localized disease in the lymph nodes, especially in the mediastinum and neck. The extra-nodal onset variant of HL is rare and has an incidence rate of 5%. NHLs occur in extra-nodal sites with much greater frequency, estimated between 10 and 35% [1,2]. NHL includes a heterogeneous group of tumors: 85–90% derive from B lymphocytes and the remaining ones arise from T lymphocytes or Natural Killer (NK) lymphocytes. Both types can have nodal and extra-nodal localization. Isaacson and Wright first described extra-nodal non-Hodgkin lymphoma (ENHL) as a distinct entity in 1984 [3]. Among NHLs in the head and neck area, B-cell NHLs are the most common, while diffuse large B-cell lymphoma (DLBCL) is the most common histological subtype. Immunohistochemistry is essential for a definitive diagnosis [4,5]. The most acknowledged risk factor for the development of ENHLs is congenital or induced immunodeficiency, such as HIV infection or Severe Combined Immunodeficiency Disease [6,7]. Epstein–Barr virus (EBV) infection seemed to be strongly associated with B-cell lymphomas in a variable number of cases [6,7,8,9,10]. Other factors implicated in NHL genesis are: genetics (e.g., family history of hematologic malignancies), immune conditions (e.g., Sjogren’s syndrome, rheumatoid arthritis), infections (e.g., HCV and Helicobacter pylori), modifiable risk factors (e.g., body mass index, alcohol consumption, and cigarette smoking), toxins and drugs (e.g., phenytoin, digoxin, pesticides), chromosomal translocations, and employment (e.g., agricultural or health workers) [9,11,12,13,14,15,16,17,18]. NHL occurs quite frequently in extra-nodal sites, and the most frequent localization is the gastrointestinal tract, followed by the head and neck area [1,2,4,5]. Oral involvement is rare, about 2% [6], and sometimes it presents as ulceration with bone destruction.

Little information is available in the literature on the characteristics of extra-nodal NHLs located in the oral cavity, as well as on comparisons of the survival rate between oral and non-oral extra-nodal NHLs in the head and neck area.

This study aims to retrospectively analyze 72 cases of head and neck ENHL in the period between 2003 and 2017 treated at the Maxillofacial Surgery Unit of the “Magna Graecia” University of Catanzaro, describing the prevalence, demographic characteristics, clinical presentations, as well as the five-year survival rate, and prognostic factors in patients, comparing oral and non-oral sites.

## 2. Materials and Methods

This retrospective study involved patients who occurred at the Maxillofacial Surgery Unit of the “Magna Graecia” University of Catanzaro in a period ranging from January 2003 to December 2017. Patients were referred to our Unit by their GPs or by the oncologists of the University Hospital.

### 2.1. Recruitment and Diagnosis

Patients recruited into this retrospective study had swelling at extra-nodal sites associated with pain, fatigue, asthenia, loss of appetite and weight, night sweats, or other nonspecific symptoms (fever, pruritus, splenomegaly, cough, and chest pain with breathing difficulties, headaches, etc.). All patients were over 18 years of age. Patients diagnosed with HL were excluded.

Subsequently, the recruited patients were divided by site into two groups: oral ENHL and non-oral ENHL.

All patients underwent laboratory tests for known risk factors (HIV, HCV, HBV, and EBV) using Doppler ultrasound study, magnetic resonance imaging (MRI) and/or computed tomography (CT) with a contrast medium to evaluate the extent of the lesion and bone involvement. Patients with salivary gland swelling underwent fine needle aspiration cytology (FNAC) or fine needle aspiration biopsy (FNAB). The first diagnostic hypothesis of these neoformations was in favor of a benign tumor of the salivary glands. The diagnosis was systematically confirmed by incisional or excisional surgical biopsy under general anesthesia. The operating samples were then sent to the Department of Pathological Anatomy for histological examination to determine the antibody expression with the immunohistochemical panel (CD3, CD5, CD10, CD20, CD23, CD79), LCA (CD45), protooncogenes BCL-2 and BCL-6, and the oncogene MUM-1. Data from the immunohistochemical panel are shown in Table 1.

### 2.2. Treatment and Follow-Up

After the histological diagnosis, all patients were referred to oncologists to receive the appropriate therapies. A bone marrow biopsy was performed to rule out tumoral infiltration. The staging was carried out according to the Ann-Arbor classification. Each patient was treated specifically with chemotherapy, and/or immunotherapy, and/or radiotherapy (RT). The most common treatment combination was the R-CHOP scheme. This scheme involved the administration of rituximab (R) with cyclophosphamide, hydroxidaunorubicin, vincristine, and prednisolone (CHOP) for six cycles. An alternative was the administration of 3–4 cycles of R-CHOP followed by RT or RT as the first line of therapy. The decision was made by a multidisciplinary team evaluating the risk/benefit ratio of each case. Patients underwent regular onco-hematological follow-ups for 5 years: every 3 months for the first year and then every 6 months.

### 2.3. Trial Procedures

This study followed the Helsinki Declaration on Medical Protocol and Ethics. The study was approved by the Ethics Committee of “Magna Graecia” University of Catanzaro (Reference number 146 of 21 May 2020) and all patients signed informed consent to be enrolled in the study.

### 2.4. Statistical Analysis

Statistical analysis was performed using the GraphPad program (GraphPad Company, San Diego, CA, USA). The Fisher exact test (χ^2^ test) was used to evaluate and compare variances (ANOVA). The p-value was then obtained: the accepted significance level was set at *p* < 0.05. The significance of the survival study was determined by performing Kaplan–Meier analysis with the log-rank test (Mantel–Cox).

## 3. Results

In total, 77 cases of primary extra-nodal lymphomas of the head and neck district were recorded in our study: 72 cases (93.5%) were NHL and 5 cases (6.5%) were HL. HLs were excluded from the study. Among ENHL patients, 29 were males (40.28%) and 43 were females (59.72%) with an average age of 64.2 years old (range 20–91 years). No patient enrolled had risk factors for lymphoma or tested positive for HIV, HCV, HBV, or EBV.

ENHLs were divided into two groups based on their localization: oral and non-oral lymphomas.

Thirty-three patients (45.83%) had an oral localization, and thirty-nine patients (54.17%) had a non-oral localization.

The most frequent oral localizations were the oral gum (n.8 cases, 24.24%) and cheek (n.7 cases, 21.21%). Other oral localizations were the palate (n.5 cases, 15.15%), jaw (n.4 cases, 12.13%), oral floor (n.3 cases, 9.09%), tonsil (n.3 cases, 9.09%), and tongue (n.3 cases, 9.09%). The most frequent non-oral localizations were the major salivary glands (74.36%): 21 cases (53.85%) at the parotid level and 8 cases (20.51%) at the submandibular level. Other non-oral localizations were the nasal cavity (n.2 cases, 5.13%), lacrimal glands (n.3 cases, 7.69%), orbital-maxillary region (n.4 cases, 10.26%), and front-temporal region (n.1 cases, 2.56%). Among ENHLs with oral localization (n.33, 45.83%), in 20 cases (60.60%), the disease presented as a well-defined mass; conversely, in 13 cases (39.40%), the disease presented as ulceration of the mucosa. Secondary bone affections were found in seven cases (21.21%). Among ENHLs with non-oral localization (n.39, 54.17%), in 35 cases (89.74%), the disease presented as a well-defined mass; conversely, in 4 cases (10.26%), the disease presented as ulceration of the skin. No secondary bone affections were found. 

ENHLs were also divided according to histology; the most represented histological type was DLBCL (n.40 cases, 55.56%). Other histological types were follicular lymphoma (FL) (n.13 cases, 18.05%), marginal zone lymphoma (MALT) (n.8 cases, 11.11%), mantle cell lymphoma (MCL) (n.3 cases, 4.16%), lymphocytic lymphoma (n.2 cases, 2.78%), Burkitt lymphoma (n.2 cases, 2.78%), T-cell lymphoma (CTLC) (n.2 cases, 2.78%), and plasmoblastic lymphoma (n.2 cases, 2.78%). Among these, 33 patients (45.83%) had an oral localization and 39 patients (54.17%) had a non-oral localization. 

Data regarding histology and localization of oral and non-oral ENHL are summarized in Table 2 and Table 3.

All patients were referred to oncologists to receive appropriate therapies; all patients received classic R-CHOP treatment with or without consolidation RT.

Fifty-six (77.78%) of the seventy-two patients considered survived at the 5-year follow-up, and sixteen (22.22%) did not, as shown in Table 4. Among these, 19 patients (33.93%) presented oral extra-nodal lymphoma; conversely, 37 patients (66.07%) presented non-oral extra-nodal lymphoma. Fifty-three patients (73.61%) presented a complete remission of the disease.

The highest death rate was recorded in oral DLBCL patients: 13 deaths (81.25%), as shown in Table 2. Sixteen patients did not reach the 5-year follow-up: 13 patients (16.88%) with oral DLBCL, 1 patient (1.3%) with oral Burkitt’s lymphoma, 1 patient (1.3%) with non-oral follicular lymphoma, and 1 patient (1.3%) with non-oral cutaneous T-cell lymphoma. The death rate was 76.47% for oral DLBCL, 50% for oral BL and non-oral CTLC, and 12.5% for non-oral FL. Considering the localization of the lymphoma, 14 patients (87.5%) died from oral disease and only 2 (12.5%) died from non-oral localization.

The Fisher’s exact test (χ^2^ test) was applied to the two groups of ENHLs, showing a statistically significant difference in the death of two groups (oral and non-oral localization) with a *p*-value = 0.0002. The death rate in the oral localization group is 40% and the death rate in the non-oral localization group is 4.76%.

**Figure 1:**
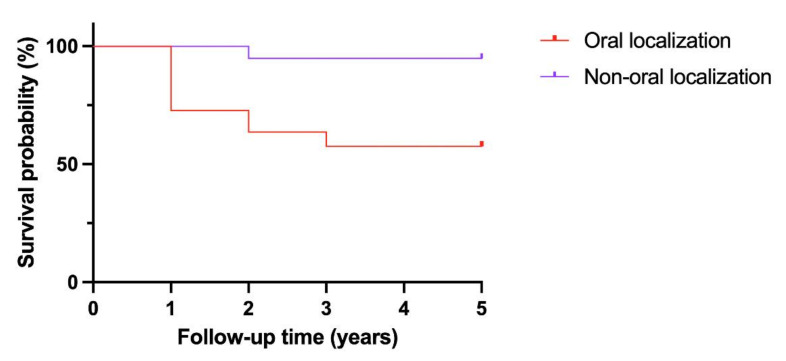
Kaplan–Meier analysis.

Kaplan–Meier analysis found the tumoral location to be a significant prognostic indicator, as shown in Figure 1. Non-oral tumoral locations showed, in fact, better survival. Cox univariate regression analysis indicated that oral versus non-oral localization showed statistical significance with a *p*-value = 0.0001.

## 4. Discussion

Lymphomas are the most common non-epithelial tumors of the head and neck area and, for this reason, maxillofacial surgeons are often involved in their diagnosis. Numerous studies in the literature have analyzed the clinical–pathological characteristics of lymphomas affecting the head and neck area. The oral cavity specifically constitutes 2% of the extra-nodal localization of NHLs [19]. ENHL affects patients in the 6th–7th decade of life, with a mean age > 65 years [20,21,22], and these data coincide with what was found in our sample. No patient presented risk factors for the onset of lymphoma or tested positive for HIV and/or EBV and/or hepatitis viruses. It is interesting to underline these last data: it is not necessary to exclude a priori an ENHL in the differential diagnosis of nodules in the head–neck and oral cavity, even in the absence of the aforementioned risk factors.

In studies reported in the literature, it has been found that lymphomas of the oral and maxillofacial regions are more common in males than in females [7,23,24]. However, none showed significant sex predilection. In the proposed study, a slight prevalence of females was also found compared to males (59.7% vs. 40.3%, respectively). The most common localization of ENHL was non-oral (39 cases, 54.17%), precisely at the level of the major salivary glands (parotid in 21 cases, 53.85%, and submandibular gland in 8 cases, 20.51%). In the literature, the most common clinical histological type is DLBCL [20,25]; it represents 68% of lymphomas affecting the oral and maxillofacial region [7]. In our study, DLBCLs are the most common histological subtype of ENHL (40 cases, 55.56%), followed by FL (13 cases, 18.05%).

The immunohistochemical analysis performed, following the panel shown in Table 1, is important for the definitive diagnosis, since it excludes other malignant diseases. For example, the CD20 test is generally positive for DLBCL but negative for anaplastic large cell lymphoma.

In our study, patients with DLBCL showed positivity for CD10 and CD20 markers and negativity for CD3 epithelial markers. Patients with FL (the second-most-frequent subtype) expressed positivity for line B markers (CD19 and CD20) and, in the majority of cases, for CD10 and BCL6, and negativity for CD5 and CD23.

Regarding prognostic factors, age, stage, and state of health are important. Some studies show that patients over the age of 60, with higher stage and/or poor health, have a higher mortality rate [26]. Patients with high-grade ENHL have a 5-year survival rate of approximately 14%, which reaches 85% for patients with an intermediate grade [27]. All patients enrolled in the study presented at stages 1-2 of the Arbor classification.

Furthermore, it was found that ENHLs are much more aggressive when they arise with an oral localization. Sixteen patients did not reach the 5th year of follow-up and, among these, fourteen had oral ENHL (87.5%) and two had non-oral ENHL (12.5%). Specifically, 13 patients had oral DLBCL, 1 patient had oral Linfoma di Burkitt, 1 patient had non-oral FL, and 1 patient had non-oral CTCL. Although the most frequent localization of lymphomas is in the major salivary glands (40.26%—22 cases in the parotid gland and 9 cases in the submandibular gland), non-oral localization seemed to be less aggressive than the oral one, with a statistically significant difference in the mortality of the two groups considered (oral and non-oral localization) with a *p*-value = 0.0002. 

In our study, the Kaplan–Meier analysis indicated that oral versus non-oral position was a significant prognostic indicator, and non-oral patients had better survival. Cox univariate regression analysis indicated that oral versus non-oral localization showed statistical significance with a *p*-value = 0.0001.

The mortality rate in the oral localization group is 40%, in contrast to the mortality rate in the non-oral localization group, which is 4.76%. 

Indeed, the highest death rate was recorded in those patients with oral DLBCL with 13 deaths (81.25%).

Generally, the 5-year survival rate for head and neck lymphomas ranges from 35.1% to 92% [28]. Regarding the prognostic factors in patients with head and neck lymphomas, the clinical stage and the response to chemotherapy are certainly the most common predictors of survival. Others may be the size of the tumor, the histological grade, the immunophenotype, the age of the patient, and the presence of viral infections.

In this study, the most significant prognostic factor was oral versus non-oral localization, with a *p*-value < 0.05. At 5 years, patients with non-oral localized ENHL showed a better response to treatment and longer survival. Conversely, patients with orally localized ENHL had less response to treatment and greater disease progression (stage progression and bone marrow disease), resulting in lower survival.

## 5. Conclusions

ENHLs are rare and their diagnosis is difficult: they are often misdiagnosed for other benign conditions (e.g., infections) or, much more frequently, malignancies (e.g., squamous cell carcinoma) [29,30]. Oral involvement of ENHLs is rare (around 2%) and it is more aggressive and has a higher mortality. A Kaplan–Meier analysis with the log-rank test was performed to determine the significance of the survival study. In addition, Fisher’s exact test was applied to the two groups (oral and non-oral location). Both showed a statistically significant difference in death with a *p*-value of 0.0001 and 0.0002, respectively. In our study, ENHLs with oral localization are much more aggressive than those with non-oral localization, with a death rate of 40% (versus 4.76 for non-oral one). The involvement of maxillofacial surgeons in the diagnosis of ENHLs optimizes diagnostic management.

## Figures and Tables

**Table 1:** Immunohistochemical panel of ENHL subtypes.

	CD3	CD5	CD10	CD20	CD23	CD79	BCL-2	BCL-6	MUM-1
DLBCL	−	−/+	+	+	−	+		+/−	+/−
FL	−	−	+	+	−/+	+	+	+	−
MCL	−	+	+	+	−	+	+	−	
MALT	−	−	−	+	−	+	+	−	
Plasmablastic lymphoma	−	−	+	−/+		−			
Lymphocytic lymphoma	−	+	+	−	+	−	+	−	+
BL	−	−	−	+	−	+	+		−
CTLC	+		−	−		−		−	

DLBCL: diffuse large B-cell lymphoma; FL: follicular lymphoma; MCL: mantle cell lymphoma; MALT: marginal zone lymphoma; BL: Burkitt’s Lymphoma; CTLC: cutaneous T-cell lymphomas.

**Table 2:** Histology and localization of oral ENHLs.

	Palate	Oral Floor	Oral Gum	Cheek	Palatine Tonsil	Tongue	Jaw	TOTAL
DLBCL	2	2	4	4	1	2	2	17 (23.61%)
FL	0	0	0	2	1	0	1	4 (5.56%)
MCL	0	0	1	0	0	0	0	1 (1.39%)
MALT	3	1	1	1	1	1	0	8 (11.11%)
Plasmablastic lymphoma	0	0	1	0	0	0	0	1 (1.39%)
Lymphocytic lymphoma	0	0	1	0	0	0	0	1 (1.39%)
Burkitt’s lymphoma	0	0	0	0	0	0	1	1 (1.39%)
CTLC	0	0	0	0	0	0	0	0
TOTAL	5 (15.15%)	3 (9.09%)	8 (24.24%)	7 (21.21%)	3 (9.09%)	3 (9.09%)	4(12.13%)	33 (45.83%)

**Table 3:** Histology and localization of non-oral ENHLs.

	Parotid Gland	Submandibular Gland	Gland Lacrimal	Nasal Cavity	Orbito Maxillary Region	Front Temporal Region	TOTAL
DLBCL	14	4	1	1	2	1	23 (31.94%)
FL	4	1	1	1	2	0	9 (12.99%)
MCL	1	1	0	0	0	0	2 (2.78%)
MALT	0	0	0	0	0	0	0
Plasmablastic lymphoma	0	0	1	0	0	0	1 (1.39%)
Lymphocytic lymphoma	0	1	0	0	0	0	1 (1.39%)
Burkitt’s lymphoma	1	0	0	0	0	0	1 (1.39%)
CTLC	1	1	0	0		0	2 (2.78%)
TOTAL	21 (53.85%)	8 (20.51%)	3 (7.69%)	2 (5.13%)	4 (10.26%)	1 (2.56%)	39 (54.17%)

**Table 4:** Five-year follow-up survival of oral and non-oral ENHLs.

	Oral Localization ENHLs Total: 33		Non-oral Localization ENHLs Total: 39	
	Surviving Patients	Non-Surviving Patients	Surviving Patients	Non-Surviving Patients
DLBCL	4 (21.05%)	13 (92.86%)	23 (57.5%)	-
FL	4 (21.05%)	-	8 (20%)	1 (50%)
MCL	1 (5.26%)	-	2(5%)	-
MALT	8 (42.12%)	-	0	-
Plasmablastic lymphoma	1 (5.26%)	-	1 (2.7%)	-
Lymphocytic lymphoma	1 (5.26%)	-	1 (2.7%)	-
Burkitt’s lymphoma	0	1 (7.14%)	1 (2.7%)	-
CTLC	0	-	1 (2.7%)	1 (50%)
TOTAL	19 (57.58%)	14 (42.42%)	37 (94.87%)	2 (5.13%)

## Data Availability

The data presented in this study are available on request from the corresponding author.
